# The tempo and mode of the taxonomic correction process: How taxonomists have corrected and recorrected North American bird species over the last 127 years

**DOI:** 10.1371/journal.pone.0195736

**Published:** 2018-04-19

**Authors:** Gaurav Vaidya, Denis Lepage, Robert Guralnick

**Affiliations:** 1 Department of Ecology and Evolutionary Biology, University of Colorado Boulder, Boulder, Colorado, United States of America; 2 Bird Studies Canada, Port Rowan, Ontario, Canada; 3 Department of Natural History and the Florida Museum of Natural History, University of Florida, Gainesville, Florida, United States of America; Arizona State University, UNITED STATES

## Abstract

While studies of taxonomy usually focus on species description, there is also a taxonomic correction process that retests and updates existing species circumscriptions on the basis of new evidence. These corrections may themselves be subsequently retested and recorrected. We studied this correction process by using the *Check-List of North and Middle American Birds*, a well-known taxonomic checklist that spans 130 years. We identified 142 lumps and 95 splits across sixty-three versions of the *Check-List* and found that while lumping rates have markedly decreased since the 1970s, splitting rates are accelerating. We found that 74% of North American bird species recognized today have never been corrected (i.e., lumped or split) over the period of the checklist, while 16% have been corrected exactly once and 10% have been corrected twice or more. Since North American bird species are known to have been extensively lumped in the first half of the 20^th^ century with the advent of the biological species concept, we determined whether most splits seen today were the result of those lumps being recorrected. We found that 5% of lumps and 23% of splits fully reverted previous corrections, while a further 3% of lumps and 13% of splits are partial reversions. These results show a taxonomic correction process with moderate levels of recorrection, particularly of previous lumps. However, 81% of corrections do not revert any previous corrections, suggesting that the majority result in novel circumscriptions not previously recognized by the *Check-List*. We could find no order or family with a significantly higher rate of correction than any other, but twenty-two genera as currently recognized by the AOU do have significantly higher rates than others. Given the currently accelerating rate of splitting, prediction of the end-point of the taxonomic recorrection process is difficult, and many entirely new taxonomic concepts are still being, and likely will continue to be, proposed and further tested.

## Introduction

The goal of taxonomy is to provide a complete, accurate catalogue of planetary biodiversity. When taxonomists encounter vouchers or exemplars of a putative new species, they collect evidence to support the hypothesis that it is distinct enough from any known species to necessitate its own name. If so, this species is formally described, is associated with a new species hypothesis, and is given a new name under the appropriate codes of nomenclature [1,2]. Over 16,000 species have been described every year between 2000 and 2010 [3], and both the number of new descriptions and the number of authors involved in species description across multiple plant and animal groups have been rising since the 1750s, while the number of species described by each author has been falling [4,5]. These observations may suggest that more taxonomists are chasing fewer remaining species, and thus species description may be approaching completion in some groups [6]. But the taxonomic process remains incomplete even after all species have been described: an unknown number of species hypotheses will eventually be re-tested and, if falsified, may be rejected in favor of other hypotheses of conspecificity [7]. The proportion of species hypotheses that will eventually be falsified may be expected to vary over time as techniques and species delimitation philosophies change and as more evidence accumulates. While much attention has been given to the description of species and higher taxa, the subsequent correction process remains understudied by comparison.

Taxonomic changes have a practical impact on lists of recognized species widely used in biological analyses [8]. In particular, there has been a sharp increase in the number of subspecies being raised to full species across a wide range of animal groups in the last few decades [9], including primates [10,11], amphibians [8], bovids [12] and birds [13]. This phenomenon has been termed “taxonomic inflation” by Isaac *et al*. [10]. Some scientists have argued that this may be the result of a shift in taxonomic practice, either from the biological species concept to the phylogenetic species concept [10] or from an assumption of free interbreeding to an assumption of reproductive isolation [14]. Focusing on birds, a recent paper has estimated that the number of globally recognized bird species may double as a result of changing species concepts and the application of molecular methods [15]. Sangster established that diagnosability rather than reproductive isolation has remained the most commonly used criterion to justify proposed taxonomic changes since the 1950s by analyzing published bird taxonomic proposals between 1950 and 2009 [13,16]. While studies of taxonomic proposals can provide valuable information on the changes being advocated by taxonomists, they do not provide information on if and when these changes became broadly recognized within the taxonomic community, and whether they were subsequently reverted. It is this perspective on the shifting taxonomic view that we attempt to measure in this article.

Simply counting the number of taxonomic changes that are recognized is not enough, as these changes may themselves require correction. Remsen Jr noted in 2015 [17] that “virtually all current systematists, regardless of species concepts, recognize that current species limits in many bird groups are far too broad, incorrect, or weakly justified”, and posited that “overapplication of Biological Species Concept (BSC) criteria by many taxonomists in the mid-20th century, often without explicit rationale, demoted by mere pen strokes hundreds of taxa from the rank of species to subspecies, before the importance of vocal differences was recognized”. Some systematists in the 1920s and 1930s were equally skeptical about demoting species to subspecies [18–21]. This all points to a current, ongoing taxonomic recorrection process, in which corrections made in the first half of the 20th century are now being reverted in light of new evidence and better tools. We delineate focused, testable questions related to this recorrection process below, but first discuss the importance of checklists for examining this recorrection process over long periods of time.

### Tracking the recorrection process using taxonomic checklists

Taxonomic changes are proposed and published in a wide variety of scientific literature, from scientific monographs to taxonomic checklists to general-interest identification guides. Previous analyses have surveyed a set of journals where taxonomic corrections are likely to be published (e.g. [13,16]), but there is no easy way to determine whether or not a particular proposal has gained traction within its taxonomic community. Conventional methods to gauge the impact of a publication, such as citations counts, do not help: a contentious proposal may be heavily cited by scientists disputing it, while a generally accepted proposal may only be cited a few times before being incorporated into compiled resources, which may then be cited instead.

Taxonomic checklists provide us with a source of taxonomic changes that are representative of a taxonomic group and are generally recognized by both taxonomists and other biologists when studying well-known taxa, such as birds. These are expert-curated authoritative lists of recognized species within a taxonomic group in a particular geographical area. Checklists are neither universally used nor necessarily congruent: different biologists often disagree on which taxonomic checklists they use when identifying taxa, and checklists may circumscribe species differently on the basis of differences in available evidence, taxonomic philosophy or tools used [22]. Taxonomic checklists may be critiqued by taxonomists [12,17] and have been used to estimate the stability of binomial names [23,24]. In this study, we focused on one such checklist project, which has been maintained over the last 130 years by the North American Classification Committee of the American Ornithologists' Union (AOU): the *Check-List of North American Birds*, hereafter referred to as the "AOU Checklist". This checklist was first published in 1886, and since then has been updated in six major and fifty-seven minor updates through 2016 [25]. The North American Classification Committee reviews corrections submitted to it based on changes proposed in the literature, and accepts those supported by two-thirds of its members [26]. These corrections are then published as a series of editions and supplements. The first update was published in 1889, yielding 127 years of corrections until 2016. The last complete edition (the 7th edition) was published in 1998 [27]. Supplements have been published at an average of one every 2.03 years. Since 2002, updates have been published every year. A subset of these changes, from 1950 to 2009, have been previously analyzed by Sangster as part of a larger study of taxonomic proposals made against global bird species in order to examine the criteria used to determine whether the rank of a species or subspecies should be changed [13,16]. Our analysis asks different questions and includes changes made to the AOU Checklist extending back to 1889, the first year in which an update to the AOU Checklist was published.

The AOU Checklist therefore provides a community review process for taxonomic corrections. It continues to be widely used as an authoritative source for taxonomic names among both professional ornithologists and an often highly engaged public, the birding community, either directly or indirectly through birding organizations and field guides that track the AOU Checklist. These include the National Audubon Society’s Bird Guide App [28], the Cornell Lab of Ornithology’s eBird/Clements Checklist [29], the American Birding Association Checklist [30], and the Sibley Guide to Birds [31].

Species description in North American birds is largely considered to be close to completion [32] after over 250 years of study [33], but the number of currently recognized North and Middle American bird species is increasing rapidly as previously described species are being recognized again. The AOU Checklist has grown from approx. 1,908 species in 1983 [34] to 2,127 species in 2016 [25], an 11.5% increase within a consistent geographical area. Since birds have been central to the development of the biological species concept [35], the phylogenetic species concept [36], as well as Remsen Jr’s observations of past, potentially problematic corrections mentioned earlier [17], they are a particularly apt group to examine the taxonomic correction and recorrection processes.

### Key questions and specific hypotheses

Our work here focusses on corrections that alter the circumscription of a scientific name without altering the name itself [37]. These are of two kinds: the division of putative species into multiple species (“splits”), which usually occurs through the raising of a subspecies to a full species, and the union of putative species into a single species (“lumps”). We interpret “species” here to mean a particular named species hypothesis recognized in a contemporary AOU Checklist, consisting of both a taxonomic name and an associated taxonomic circumscription. In other words, we consider a taxon to be a species if a biologist relying on the most recently published AOU Checklist would have considered it to be a species, using no other information from other sources. Another possible definition of a species, as a taxon consisting of a set of clearly-defined subspecies, might have been used before the sixth edition of the AOU Checklist, published in 1983 [34], but after this date the AOU Checklist published lists of recognized species only, and no longer provide a comprehensive list of the subspecies recognized within each species.

In order to understand how taxonomic circumscriptions change after initial description, we quantify several rates. We define the “correction rate” as the proportion of currently recognized species that have ever been corrected, and the “recorrection rate” as the proportion of currently recognized species that have been corrected more than once. The “full reversion rate” is the proportion of all corrections that completely reverted an earlier correction (i.e. when a lump is subsequently resplit, or a split is subsequently relumped). Note that full reversions may not yield exactly the same circumscriptions. We further define a more general “reversion rate” as the proportion of all corrections that have been partially or completely reverted, in which two or more split species are relumped or where two or more lumped species are resplit, along with other sister species. These rates are similar to Alroy’s rates of invalidation and revalidation [38], but applied to currently recognized species and taxonomic changes rather than to taxonomic names. To quantify how these taxonomic corrections led to the current taxonomy, we summarized the sequence of lumps and splits that involve each of the currently recognized species.

In coining the term “correction rate”, we are not implying that every change made to a taxonomic checklist will eventually be judged correct. Instead, our use of terms recognizes that every change in delimitation is made with the intention of improving the accuracy of the checklist by correcting previous issues. By doing so, we are not making quality judgements on the corrections and their subsequent recorrections. Rather, we are focusing on the pattern of correction and recorrection we observe, which are ultimately indicative of taxonomic progress. We decided not to refer to these as “changes”, as that includes all changes that might be made to a taxonomic checklist: changes in spelling, in authorship, in higher taxonomy or even in common names. We also considered using the term “revision”, but decided that it might be confused with “taxonomic revisions”.

To test whether newly recognized bird species were the result of resplitting previous lumps, we first determined the proportion of all splits that were the result of a previous lump and then tested whether lumps were as likely to be reverted as splits were. If this period of splitting is largely the result of undoing lumping from before 1980, we would expect to see many more splits reverting previous lumps than vice versa. If, on the other hand, most splits are unconnected with previous lumps, this suggests taxonomists are generating novel circumscriptions and not solely correcting a backlog of incorrect lumping. We also ask if certain bird groups, at multiple taxonomic hierarchical levels, are more likely to be corrected than others, given that traits that make species delimitation more difficult may be shared among closely related species. For instance, some traits may make species boundaries more difficult to identify or by making the species themselves harder to study. Our analyses thus provide insight into past and current taxonomic correction processes for North American birds, especially how often entirely new concepts have been and are still forming as opposed to the re-recognition of previously subsumed concepts.

## Materials and methods

### Source data

The AOU Checklist consists of sixty-four checklists published between 1886 and 2016: seven major editions, which list every recognized species, and fifty-seven “supplements”, which list changes to the checklist since the previous supplement (S1 Table). We began with lists of additions, deletions and changes in scientific names to the AOU Checklist collected by one of the authors (DL) for checklists published between 1886 and 2012. These changes were collected as part of the online database Avibase [39], which also contains information on which circumscriptions are entirely contained within others [22]. Based on this information, we excluded additions and deletions that did not involve intersecting or overlapping species circumscriptions for recognized species–in most cases, these were the results of changes in distributional records, such as when a previously described species was discovered in North America. We checked changes involving overlapping circumscriptions against the AOU Checklists themselves to identify those that were explicitly stated to be a lump or split in the publications; for instance, " …we divide *B[ranta] canadensis* by recognizing a set of smaller-bodied forms as the species *B*. *hutchinsii* …" from the 45th supplement [40]. Lumps or splits identified by Avibase were excluded from our analyses if the AOU Checklist did not explicitly indicate them as such, since Avibase may have made this determination based on the view of later taxonomists while we aimed to capture the contemporary view as far as possible in order to closely track changing bird taxonomy as recorded by the AOU Checklist. As a result, our measures are conservative counts that are likely smaller than the true values–a more thorough study of the contemporary literature might lead to evidence that a particular addition was known at the time to be a split. Since the 34th Supplement provided a list of all species recognized in 1982 and the AOU published an online spreadsheet of recognized species in 2016, we used these to correct any discrepancies that may have entered our dataset before those dates. For checklists between 2013 and 2016, which postdate our initial export of Avibase data, we extracted the lumps, splits and name changes directly from the supplements themselves [25,41–43]. In all, we found 148 lumps and 191 splits recognized by the AOU Checklist between 1889 and 2016, covering North America excluding Hawaii before 1982 and North and Central America including Hawaii after 1982.

**Fig 1:**
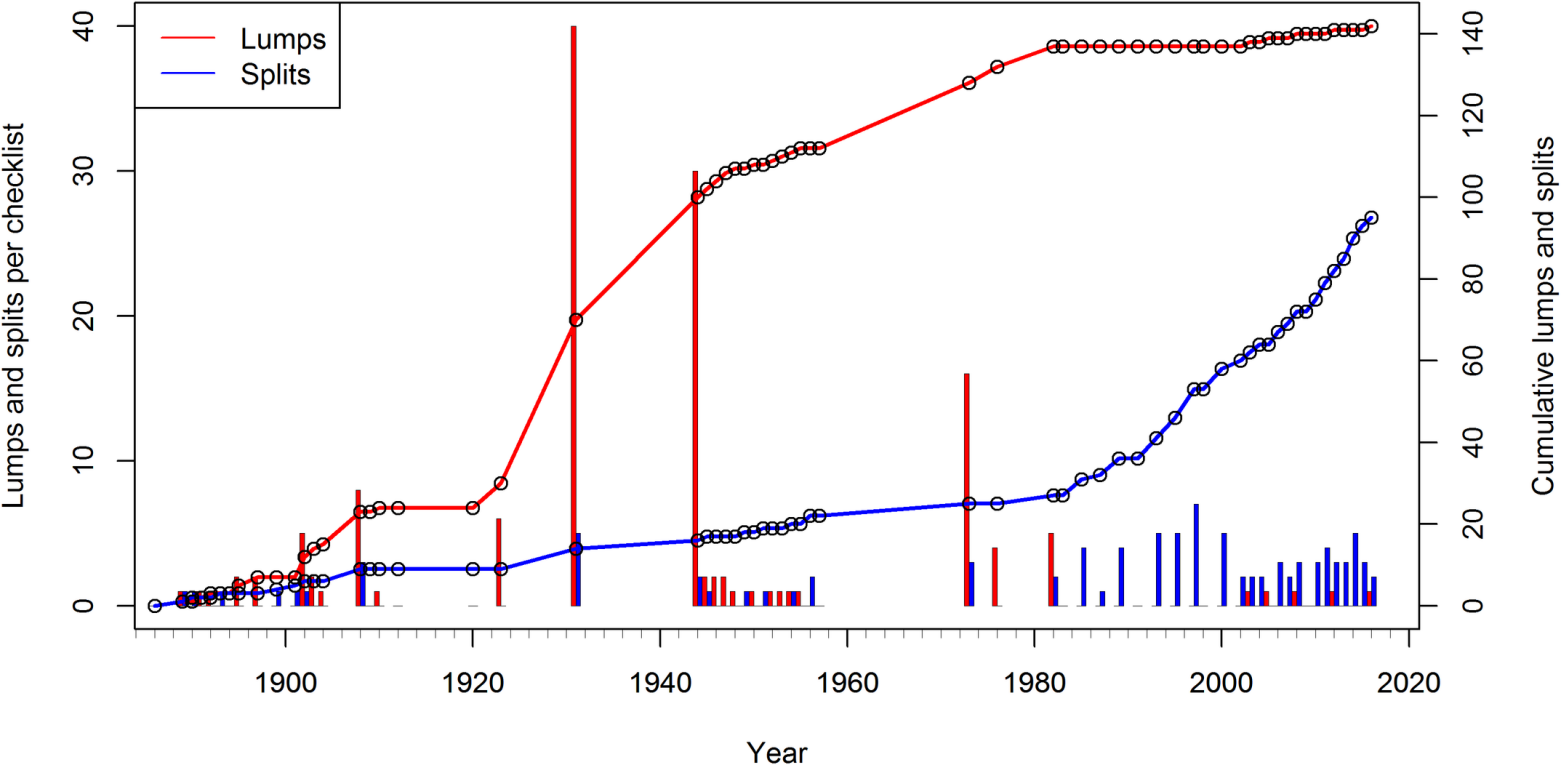
Individual and cumulative lumps and splits within the AOU Checklist between 1886 and 2016. Each circle represents a single checklist, showing periods of activity (1944–1957, 1980–2016) as well as periods of relative inactivity (1920s and 1960s).

**Fig 2:**
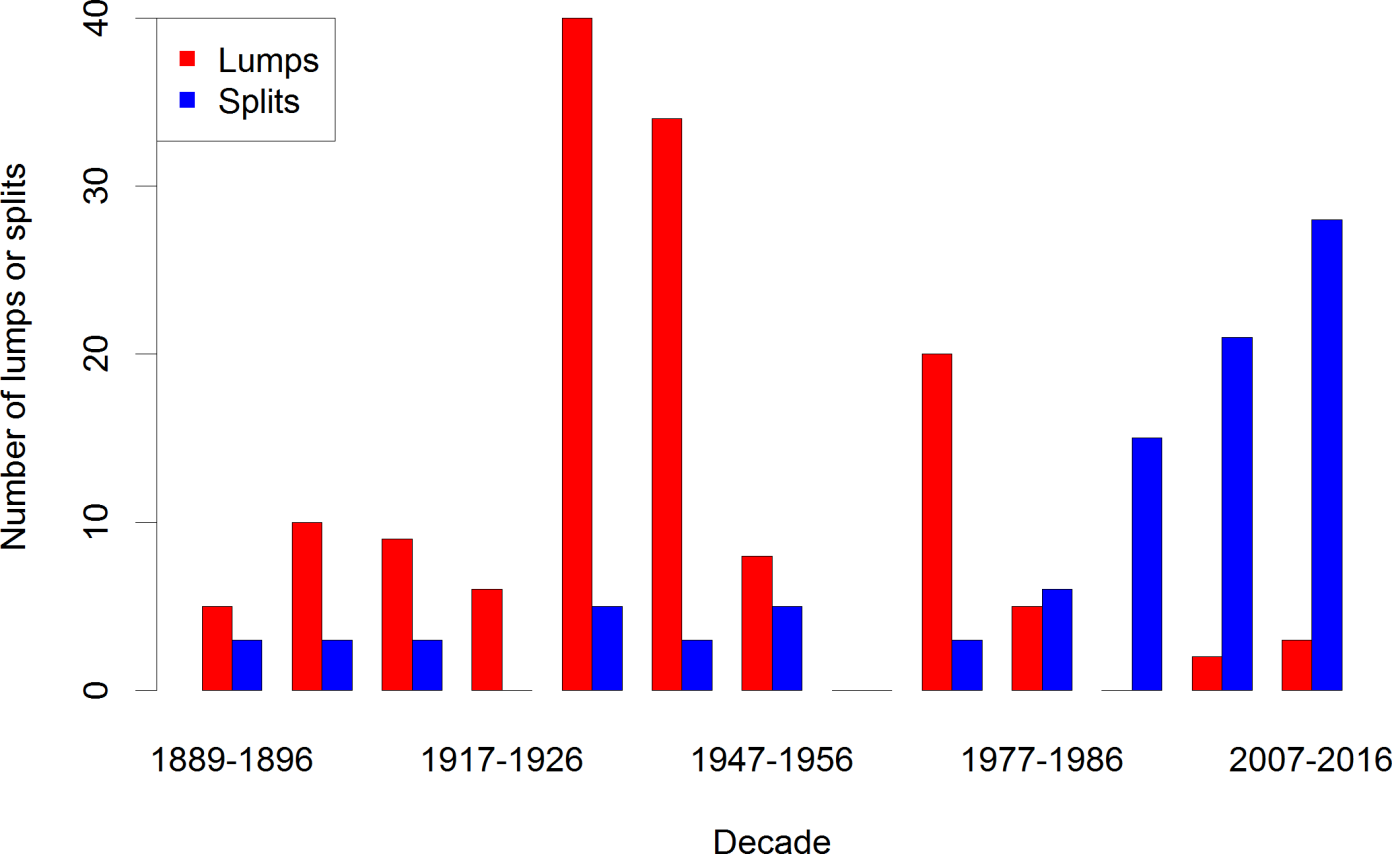
Bar plots of number of lumps and splits by decade showing accelerating number of splits per decade in the present. Note that the first decade is incomplete, as we only have data on the eight years from 1889 to 1896.

Our analysis was complicated by a large increase in the geographic range of the AOU Checklist in 1982 and 1983, expanding to include Mexico, the Hawaiian Islands, the Caribbean Islands and Central America while removing species found only in Greenland. From approx. 858 species recognized in the 33rd Supplement (1976) [44], the number of recognized species rose to 937 species in the 34th Supplement (1982) [45] and to approx. 1,908 species in the 6th Edition (1983) [34] (S1 Table). To obtain a consistent picture of taxonomic corrections over as long a time period as possible, we eliminated all additions, deletions, renames, lumps and splits involving species first added to the checklist after 1981, thus isolating corrections among species in continental North America. This resulted in 142 unambiguous lumps and 95 unambiguous splits recognized by the AOU Checklist between 1889 and 2016 (S2 Table). After eliminating these changes, the number of recognized species varied from 771 (in 1886) to 875 (in 1956), before reaching its current count of 851 species in 2016 (S3 Table). Of these 851 recognized species, 17 were the result of “extralimital” lumps and splits that took place outside of the AOU Checklist’s geographical area, resulting in 834 currently recognized species after filtering. We eliminated ten checklists because no unambiguous lumps or splits took place in them (1894, 1909, 1912, 1920, 1957, 1983, 1991, 1998 and 2009). We calculated the cumulative change in the number of lumps and splits over the last 127 years (Fig 1) and summarized these changes by decade to look at overall trends (Fig 2).

To account for synonymy while measuring these rates, we assembled “name clusters” that link together species names that have been renamed. For example, *Phyllopseustes borealis* was first added to the AOU Checklist in 1886, but has since become known as *Acanthopneuste borealis* and *Phylloscopus borealis* as it was moved between different genera. These three names constitute a single name cluster, and a lump involving one name will be matched in our analysis with a split involving another name in the same name cluster. All 834 name clusters are included in S3 Table, where extralimital name clusters are indicated by an ‘NA’ in the ‘Order’ column.

This approach can be contrasted with a “taxonomic concept”-based approach. Such an approach might use the vocabulary established by Franz and Peet [46] to identify precise relationships between different taxonomic circumscriptions, even when these circumscriptions are identically named (e.g. *Branta canadensis* published in the AOU Checklist before and after 2004). However, doing so would require reconstructing the relationship between these circumscriptions as understood at a particular point in time, which is challenging to do comprehensively, accurately and consistently over a 127 year period. Instead, we opted to document name clusters being lumped or split as well as the name clusters resulting from the change. This simpler model provides a way to compare taxonomic changes with each other between different time periods.

### Taxonomic corrections

To measure how often individual lumps and splits are reverted, we identified partial and full reversions for every lump and split. A full reversion is one where the other change exactly undoes the first one, such as *Gallinula galeata* being lumped into *Gallinula chloropus* in the 18th Supplement [47] but then resplit in the 52nd Supplement [48]. A partial reversion occurs when two or more lumped species are resplit or two or more split species are relumped along with other species. An example is *Rallus obsoletus* being lumped into *Rallus longirostris* in the 19th Supplement [49], but later resplit in the 55th Supplement [42] into *R*. *obsoletus* and *R*. *crepitans*. It is possible but not guaranteed that the circumscription for *R*. *obsoletus* as of the 55th Supplement is congruent to the circumscription for *R*. *obsoletus* before the 19th Supplement; therefore, our analysis assumes that every lump or split results in a new circumscription. The full list of reversions is included in the table of lumps and splits (S2 Table). To test whether resplitting previously lumped species directly caused increases in recognized species, we determined whether lumps were as likely to be resplit as splits were to be relumped.

For each currently recognized species name cluster, we identified the sequence of lumps and splits in which they have been involved. In particular, we wanted to know what proportion of name clusters had never been corrected, what proportion had been corrected one or more times (the “correction rate”), and what proportion had been corrected more than once (the “recorrection rate”). In order to determine the trajectory of corrections necessary to obtain the current name cluster, we tallied up the number of lumps and splits each name cluster had been involved with in chronological order. We also counted the total number of lumps and splits for each name cluster. Since every lump and split potentially results in a new circumscription (i.e. a new taxonomic concept *sensu* Franz *et al*. [50]), this gives us the number of circumscriptions associated with each species name cluster. This is included in the table of name clusters (S3 Table).

### Differences in correction rates among higher-level taxa

To determine whether different taxonomic groups showed significantly different correction rates, we modeled the number of taxonomic corrections (lumps + splits) involving currently recognized name clusters as a Poisson distribution, in which the rate at which new corrections are made to species (*λ*) is assumed to be constant within a taxonomic group. Since our analysis focuses on 834 currently recognized species clusters, we used the higher taxonomic system provided by the AOU Checklist in 2016. Our model had three hierarchical levels of grouping: at the level of genus (*π*), family (*τ*) and order (*ρ*). Additionally, we included an offset to account for the different lengths of time that different species have been in the checklist. Our hierarchical model can be described as:

$$y_{i}∼Poisson(\lambda_{i})$$

$$log(\lambda_{i})=\lambda_{0}+\pi_{i}+\tau_{j[i]}+\rho_{k[j[i]]}+log(t_{i})$$

Each of these parameters were modeled as normally distributed random variables, with a mean of zero and with variable standard deviations (*σ*_*π*_, *σ*_*τ*_ and *σ*_*ρ*_ respectively). The terms refer to the individual (*λ_i_*), the genus the individual belong to (*π_i_*), the family the genus belongs to (*τ*_*j*[*i*]_) and the order the family belongs to (*ρ*_*k*[*j*[*i*]]_). *t*_*i*_ is the number of checklists that this species has been recognized in the AOU Checklist, to control for some species having been recognized by the AOU Checklist earlier, giving them a longer time span within which to be lumped or split than others. This model failed to converge in rSTAN 2.15.1 [51], and so we used transformed parameters to define standard normal deviations that were multiplied by the variable standard deviations (see S1 Code). This model converged successfully in rSTAN and gave us an estimate of the overall mean rate of correction (*λ*) as well as the mean rate for every order (S4 Table), family (S5 Table) and genus (S6 Table).

## Results

### Overall trends in lumping and splitting

Currently, the AOU Checklist recognizes 2,127 species from North and Central America, including Hawaii [25]. The rate of species description among these species has been falling steadily: 191 species (9%) have been described since the AOU Checklist was first published in 1886, half of which (101 species or 4.8%) have been described since 1900, and only 14 species (0.7%) have been described since 1950. When we looked at the 834 species remaining in our checklist after filtering out names added after 1981 as well as extralimital species, 30 (3.6%) were described since 1886, 15 (1.8%) since 1900 and only three species (0.4%) since 1950. Thus, primary species description in this group appears to be proceeding at a very low but non-zero rate.

In contrast, taxonomic corrections have been proceeding at a rapid rate: we discovered 142 unambiguous lumps and 95 unambiguous splits on species name clusters added before 1982. Examining the cadence of lumping and splitting (Fig 1), we note large numbers of lumps, in particular the 40 lumps in the 4th edition in 1931 [52], 30 lumps in the 19th supplement in 1944 [49], and 16 lumps in the 32nd supplement in 1973 [53]. While there are no specific spikes in the number of splits, most of the splits (70, or 73.7%) in our dataset took place in or after 1980. Cumulative plots show that lumping has all but ceased since 1980, while splitting rates have sharply increased since the 1980s and continue to accelerate to the present day (Fig 2). Based on the trends in the data, new formation of taxonomic concepts in North American birds since 1950 and particularly since 1980 is mainly driven by splitting of taxa. As noted by Gill [14] and Barrowclough et al. [15], the era of splitting appears to be far from over.

### Full and partial reversions

We begin by considering the corrections themselves to determine the scope of original correction and subsequent recorrection. We found a total of 142 lumps and 95 splits occurring amongst currently recognized species that were first added to the AOU Checklist before 1982. Of these, 7 lumps (4.9%) and 22 splits (23.2%) fully revert a previous split or lump, respectively, for an overall reversion rate of 12.2%. If we count both full and partial reversions, these numbers increase to 12 lumps (8.5%) and 34 splits (35.8%) partially reverting an earlier correction, for an overall partial reversion rate of 19.4%. Thus, 80.6% of all corrections do not revert a previous correction within the AOU Checklist, and 64.2% of splits do not revert a previous lump within the AOU Checklist. There were significantly more splits than lumps both fully reverting previous corrections (exact binomial test, p < 0.01) as well as partial corrections (exact binomial test, p < 0.01). We found the proportion of splits reverting previous lumps were significantly higher than would be expected based on the ratio of lumps to splits in our dataset (Fisher’s exact test, p < 0.001). Less than half of all lumps have been partially (36 lumps, 25.4%) or fully (22 lumps, 15.5%) reverted, suggesting that the resplitting process is either mostly incomplete or that most lumps may never be resplit. It is worth emphasizing that our knowledge of which corrections were previously corrected is limited to the period of our dataset: if a period of lumping took place before the initial publication of the AOU Checklist, for example, then a higher proportion of the changes currently in our dataset might be involved in a change or revert previous changes than we report. This is an inherent limitation to our approach: we cannot improve this by increasing the coverage of our dataset, as there will always be a period of taxonomic changes before the first checklist we consider.

We can also determine the proportion of all corrections involved in any recorrection, either by correcting a previous correction or by being corrected in the future. We found 54 corrections (22.8%) involved in full reversions while 86 corrections (36.3%) were involved in partial reversions. Therefore, 63.7% of all corrections are neither correcting a previous correction nor have yet been corrected by a future correction.

### Corrections involving currently recognized species

Identifying the species affected by the corrections we have catalogued is complex: every correction affects multiple species, and species that are lumped are no longer recognized as species by the AOU Checklist. Species may no longer be recognized in the AOU Checklist if the species is no longer found within the checklist area, or may be added not for any taxonomic reason but solely because it has been introduced into the checklist area. Thus, there is no clear denominator of the total number of species recognized with which we can compare the number of species affected by taxonomic corrections.

**Fig 3:**
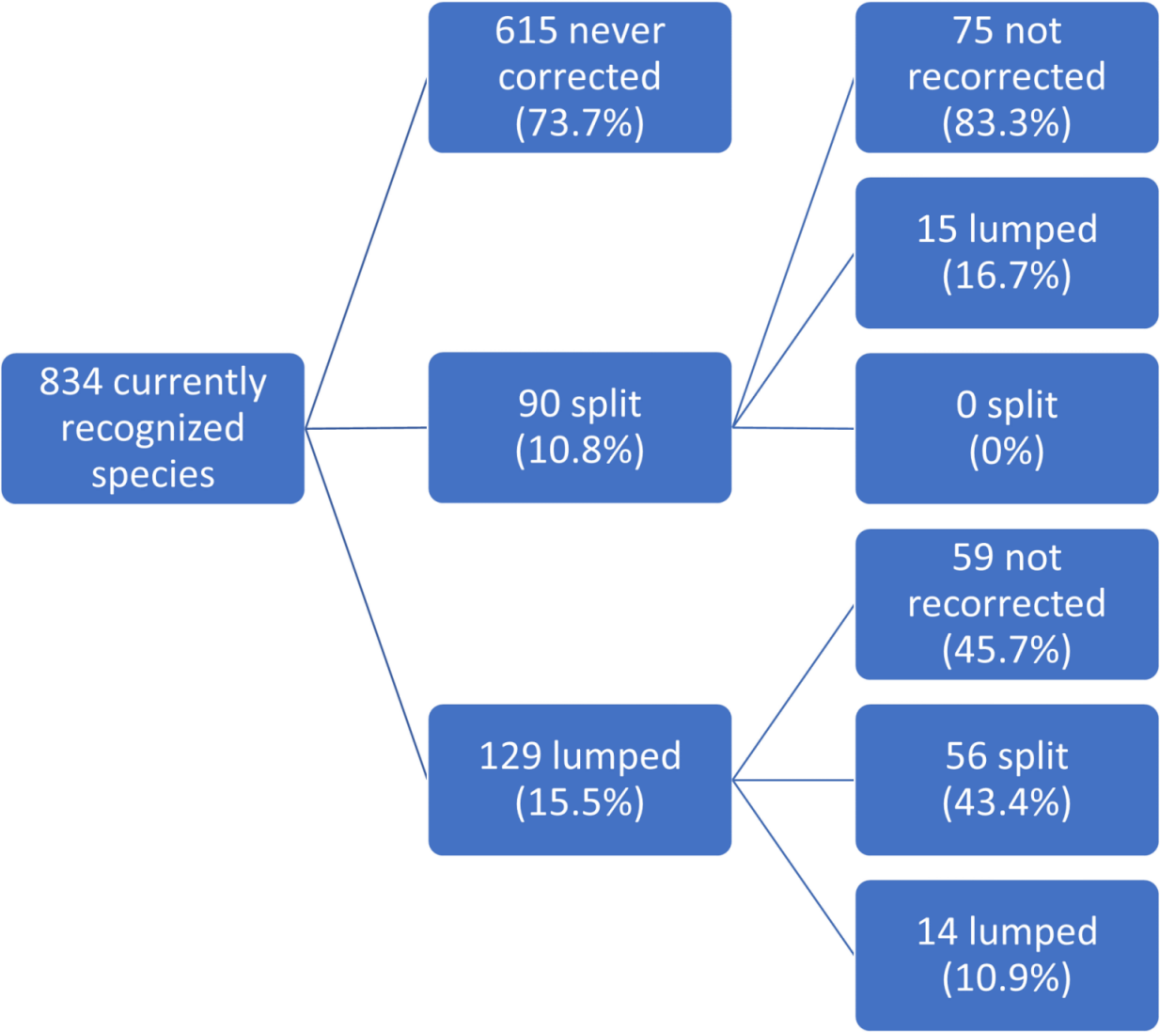
A diagrammatic representation of the corrections involved in generating the 834 currently recognized name clusters. Note that a lump followed by a split does not imply that the split reverted the lump; different species might have been split out of the lumped circumscription to obtain the current circumscription. We see relatively low rates of initial corrections, but once corrected, 43% of species involved in lumps are later involved in splits, while only 17% of species involved in splits are subsequently involved in lumps. 18 species that were involved in more than two corrections are summarized by their first two corrections above.

Instead, we focused our analysis on one particular question: if a researcher today were to use a species name currently recognized by the AOU Checklist, how likely is this to be a species that has been corrected within the lifetime of the Checklist? As previously described, to maximize the time period we could cover, we started with the 2,127 species currently recognized, eliminated species added after 1981 and obtained 834 currently recognized species names (S3 Table). Of these, 615 species (73.7%) have never been corrected in the course of the Checklist (Fig 3), suggesting that most species are not corrected over long periods of time.

To determine the sequence of lumps and splits affecting each species, we identified all lumps and splits involving the species (as either source or result) and arranged them in chronological order. Fewer than 2.2% of species were involved in more than two corrections, and so we have summarized these results on the basis of the first two corrections involving each species. Of the 219 species (26.3%) that have been corrected one or more times, more species were first lumped (129 or 58.9%) than first split (90 or 41.1%). As a reminder, these are the number of *species* that are involved in lumps and splits, not the number of corrections themselves. However, 43.4% of species involved in a lump were subsequently involved in a split, while only 16.7% of species involved in a split were subsequently involved in a lump. 85 species (10.2%) were corrected two or more times. Thus, the overall correction rate was 26.3% and the overall recorrection rate was 10.2%. 18 species that were involved in more than two corrections are summarized by their first two corrections in Fig 3, and are: *Junco hyemalis* (5 corrections); *Aphelocoma californica*, *Ammodramus caudacutus* and *Rallus crepitans* (4 corrections each); *Picoides arizonae*, *Quiscalus major*, *Dendragapus fuliginosus*, *Butorides striata*, *Branta bernicla*, *Melanitta fusca*, *Melozone crissalis*, *Ammodramus nelsoni*, *Dendragapus obscurus*, *Troglodytes hiemalis*, *Rallus obsoletus*, *Melozone fusca*, *Oceanodroma leucorhoa* and *Picoides stricklandi* (3 corrections each).

### Which species are most likely to be lumped or split?

We used a Bayesian hierarchical model to determine if some orders, families or genera were more or less likely to be associated with multiple taxonomic concepts than others among the 834 species we used in our analysis. We used the contemporary taxonomy used by the AOU Checklist in 2016 to determine order, family and genus [25]. Our model fit a Poisson distribution with *λ* = 0.3985 While no orders (S4 Table) or families (S5 Table) showed significantly higher or lower rates of correction, 22 genera recognized by the AOU Checklist in 2016 showed significantly higher rates of corrections: *Ammodramus* Swainson, 1827, *Anser* Brisson, 1760, *Aphelocoma* Cabanis, 1851, *Artemisiospiza* Klicka and Banks, 2011, *Baeolophus* Cabanis, 1850, *Branta* Scopoli, 1769, *Butorides* Blyth, 1852, *Dendragapus* Elliot, 1864, *Empidonax* Cabanis, 1855, *Gallinago* Brisson, 1760, *Gallinula* Brisson, 1760, *Junco* Wagler, 1831, *Leucosticte* Swainson, 1832, *Limnodromus* Wied, 1833, *Melanitta* Boie, 1822, *Melozone* Reichenbach, 1850, *Puffinus* Brisson, 1760, *Quiscalus* Vieillot, 1816, *Rallus* Linnaeus, 1758, *Sternula* Boie, 1822, *Sula* Brisson, 1760, and *Troglodytes* Vieillot, 1809 (S6 Table). These correspond to 6.5% of the 338 genera in our dataset and belong to fifteen families across eight orders.

## Discussion

Birds are often cited as a taxon in which species description is likely to be complete–for example, Bebber *et al*. [32] estimated on the basis of species description curves that only 26–93 bird species remained to be described. The AOU Checklist supports this pattern, with over 90% of currently recognized species having been described before the Checklist was first published in 1886, and a mere fourteen species described since 1950. Taxonomic work in this group is nevertheless incomplete. When only considering species added before 1982 to the American Ornithological Union checklist, i.e. those species that was recognized by the checklist when it was limited to North America excluding Mexico, we found 142 lumps and 95 splits which were involved in the correction of 218 currently recognized North American species (correction rate: 26.3%), of which 85 currently recognized species (recorrection rate: 10.2%) were involved in more than once correction.

We did not find a concentration of corrections in any one order or family, but 6.5% of North American bird genera in our study showed significantly higher rates of taxonomic correction. We were unable to find a higher taxonomic signal, related to shared characteristics and life-history, or any immediately obvious other factor such as size of the genus. We note, however, that these numbers only reflect a part of the complete debate over these circumscriptions, since we analyze changes within a single checklist. Thus, a species circumscription that is heavily debated in the literature may not have been recognized by the AOU Checklist until they decided collectively to support one particular interpretation. An example of this is the species *Branta hutchinsii*, which had been recognized as a subspecies of *Branta canadensis* by the AOU Checklist until it was raised to a full species in the 45th Supplement [40]. Before the AOU Checklist was first published, both its original author [54] and John James Audubon [55] treated it as a separate species, and proposals for treating it as a separate species date back until at least 1946 [56]. Thus, we re-emphasize that both the per-genus correction rates and the overall correction, recorrection and reversion rates we document reflect a conservative measure of all proposed corrections in the literature, but are likely accurate for the widely-recognized corrections that scientists use in practice. Studying taxonomic proposals directly [13,16] can provide a more detailed analysis of the corrections being advocated for and being discussed by taxonomists, but provide limited opportunities for assessing how these corrections affect the interpretation of actual data. In understanding the entirety of the taxonomic process–how a taxonomic proposal is conceived, tested, published, contested, recognized, corrected and recorrected–both of these approaches have much to contribute, and further studies towards a unified theory of taxonomy is necessary. The first step might be to collect and publish taxonomic changes from both taxonomic proposals and checklists, such as those we include (S2 Table), which might facilitate large studies covering several parts of this taxonomic process.

Our results show a clear period of lumping in the 1920s to the 1980s, followed by a period of rapid splitting in the AOU checklist. 19.4% of all lumps and splits in our dataset are full or partial reversions of a previous correction, 74% of which are splits reverting a previous lump. Reversions are clearly a part of the current period of splitting, but the vast majority (64.2%) of splits do not partially or fully revert a previous lump. Furthermore, 80.6% of all corrections do not partially or fully revert a previous correction, showing that the generation of circumscriptions novel to the AOU Checklist have been and continue to be a critical part of taxonomic revision. Both previously uncorrected species circumscriptions as well as previously recognized corrections are being actively retested and corrected by North American bird taxonomists today.

A checklist-based approach to studying taxonomic change has an inherent limitation in that it tracks only a single taxonomic view over time, and our results do not necessarily reflect the patterns we would observe if we examined other taxonomies of North American birds or in bird checklists globally. There is also no documented evidence that the AOU Checklist’s methods and philosophies have changed since at least the advent of the BSC in the 1930s: for example, the committee members “strongly and unanimously continues to endorse the biological species concept (BSC)” in 1998 [57]. Coincident have been development of concepts such as the Comprehensive Biological Species Concept in 1999 [58], which advocates for a less narrow interpretation of the BSC. Sangster’s bibliometric analysis [16] further supports the view that there has not been a major shift in philosophy or tools over the course of this checklist: he found that the majority of lumps and splits proposed for global bird species between 1950 and 2009 used diagnosability as a criterion for delimiting species, with reproductive isolation used in fewer than half the proposals in every decade (with the exception of the 1970s, when it briefly reached 50%). However, North American bird taxonomy began long before the first AOU Checklist was published–the earliest changes we observe might have corrected taxonomic opinions that were incorporated into the first edition of the Checklist, and further cycles of lumping and splitting might have been observed if the AOU Checklist extended further back in time. As we did not incorporate pre-1889 information into our study, we likely underestimate the number of changes that corrected previous changes, and overestimate the proportion of names that had never been corrected.

The stability we observe in the methodology of the AOU Checklist raises the question of possible causes of the shift from lumping to splitting in the 1980s. The 1980s were a period of great technological innovation in both biology, with the development of Sanger sequencing in 1977 and the polymerase chain reaction in 1983, and in the world at large, with the development of the personal computer in the late 1970s and early 1980s and NSFNET, the predecessor of the Internet, in 1985. The use of ancient DNA are also changing our understanding of evolutionary relationships among groups of birds [59]. Any of these, as well as any number of changes in the funding or production of taxonomic work, may have led to an increased output from taxonomists, shown as an increased rate of correction since the 1980s. We observe that rates of species description [4,5] as well as the number of scientists involved in species description [60] have been increasing since the 1950s. Whatever factors are responsible for that increase may also be increasing the number of taxonomists testing and correcting taxonomic circumscriptions, leading to the accelerating splitting rates we see. Further, some of that work appears to have been put into the recorrection of previously corrected species circumscriptions. One further line of inquiry along these lines is to focus on changes that were partially or completely reverted, and compare the evidence used to justify the initial correction with the subsequent recorrection.

Extrapolating this pattern into the future and using taxonomic concepts (sensu Franz et al. [50]) as the key unit, rather than simply the species names, we expect a continuing period in which both the development of concepts that have not been previously recognized by the AOU Checklist and the reversion of previously recognized concepts are carried out side-by-side. The refinement of theoretical approaches to species delimitation and growth in empirical datasets such as genomic data should lead to fewer novel species circumscriptions and taxonomic corrections remaining to be found. While taxonomists will likely continue to debate which corrections are accurate and which are not, we extrapolate an end state in which taxonomic corrections fall to a low, but non-zero rate, in much the same way species description rates have in North American birds. This rate will never reach exactly zero, not only because new evidence will continue to refine our view of historical speciation, but also because speciation is an ongoing process that will continue to lead to divergent lineages and thus to new species, likely at a very low rate. Species description and lumping appear today to be proceeding at these low but non-zero rates, especially considering the much higher rates they demonstrated in the 1800s and between 1930 to 1960 respectively. By comparison, splitting is proceeding at an unprecedented rate within the checklist, which continues to accelerate. If they predominantly reverted previous lumps, we might have been able to extrapolate when all previous lumps might be fully resplit, but we find that only 25% of lumps have been reverted, and 81% of all changes do not revert a previous change. Therefore, our results do not provide an empirical means to predict when this end state might be reached. However, we do note that continuing acceleration along the trajectory we show here could hasten what others [14] have argued is likely to be a slow process.

How general are the patterns we show here for other taxa and regions? Bird taxonomy was strongly impacted by extensive lumping from the 1920s to the 1980s, but we still find that the outcome of splitting is as much new taxonomic circumscriptions as it is reversions to previously recognized circumscriptions. Among other groups in which “taxonomic inflation” has been observed, such as primates [10,11], amphibians [8], bovids [12] and birds [13], we might expect to see a similar pattern of mixed taxonomic corrections and recorrections explaining the increase in the number of recognized species. More broadly and across a larger spectrum of the tree of life, we still know little about groups where current description rates far swamp any taxonomic corrections. As studies like ours are replicated, we hope that broader answers to questions about the tempo, mode and potential end-states of taxonomic discoveries can be found.

A final motivation for our work was the extent to which taxonomic correction leads to errors when biodiversity analyses use species name without considering the different circumscriptions that may be associated with that name. In our dataset, we find that 74% of species names were only associated with a single circumscription, 16% of species names were associated with exactly two circumscriptions (by being corrected once) and only 10% of species names were associated with more than two circumscriptions (by being corrected two or more times). Thus, a still significant proportion of species names are associated with multiple taxonomic concepts that make simple taxon labels ambiguous [22,37]. Errors may be minimized by focusing analysis on species known to have no taxonomic corrections, but in North American birds, no single order or family was found to be more likely to be recorrected. This suggests one simply cannot avoid "problem-areas" in North American bird groups except possibly at the generic level. Instead, any broad-scale analysis that ignores taxonomic concepts is likely to introduce some error.

Our work draws attention to the parts of the taxonomic process that are often overlooked when focusing exclusively on species description and on names without reference to circumscriptions. Large public databases of species descriptions have been published by several organizations, including the Catalogue of Life [61], Zoological Record [62], the Plazi Treatment Bank [63] and downstream databases such as BioNames [64]. These resources have facilitated many studies of the cadence of description patterns [4], changing properties of species descriptions [65] and estimates of the number of species remaining to be discovered [60]. The first databases of circumscriptions have been built, including Avibase, which formed the basis of this study [39,66] and some biodiversity databases now incorporate circumscriptions, including citizen science platforms such as iNaturalist [67]. New philosophical, ontological and software tools to identify [68], describe [46], share [69,70] and reason over [71,72] taxonomic circumscriptions have become available recently, which we believe will lead to better, shareable circumscription datasets that provide a means to move beyond simply capturing name strings and towards the more fundamental units of biodiversity. The circumscriptions we used in this project are only one interpretation of the taxonomic acts that we have studied; by making the data we used in this project available, we hope that future work will be able to build on our work to assemble larger datasets, leading to a more thorough understanding of how taxonomic corrections have refined our knowledge of global biodiversity and how they will continue to do so in the future.

## Supporting information

S1 Table List of AOU checklist updates with authors and estimated counts of recognized species. (CSV)

S2 Table List of 142 lumps and 95 splits after filtering out all changes after 1981. Includes information on all the changes that revert a particular change, as well as the subset of those reversions that are complete–where one change perfectly undoes another change. Note that “reversion” does not imply a particular ordering in time: both the initial change and all its partial or complete reversions will list the other change as reversions.
(CSV)

S3 Table 851 currently recognized species after filtering out all changes after 1981, including 17 extralimital species. Includes a count and list of taxonomic concepts associated with each name, the ‘trajectory’ of changes (the sequence of additions, deletions, renames, lumps and splits) we know about associated with this name or its synonyms and in which dataset this name and its synonyms were first added. The remaining columns are from the 2016 Checklist of North and Middle American Birds, downloaded from http://checklist.aou.org on October 3, 2016. Extralimital species, i.e. those involved in lumps and splits but not found within the geographical area of the checklist, have ‘NA’ in all higher taxonomy columns and were not present in the 2016 Checklist.
(CSV)

S4 Table Results of the hierarchical model at the order level. The total and mean number of redescriptions observed in each order are indicated. The ‘min’, ‘max’ and ‘interval_width’ values refer to the 95% credible interval around the ‘mean’ for the log difference in the λ attributable to that order. The lower interval is greater than zero where the order has a significantly higher rate of taxonomic redescription than other orders.
(CSV)

S5 Table Results of the hierarchical model at the family level. The total and mean number of redescriptions observed in that family are indicated. The ‘min’, ‘max’ and ‘interval_width’ values refer to the 95% credible interval around the ‘mean’ for the log difference in the λ attributable to that family. The lower interval is greater than zero where a family has a significantly higher rate of taxonomic redescription than other families.
(CSV)

S6 Table Results of the hierarchical model at the genus level. The total and mean number of redescriptions observed in that genus are indicated. The ‘min’, ‘max’ and ‘interval_width’ values refer to the 95% credible interval around the ‘mean’ for the log difference in the λ attributable to that genus. The lower interval is greater than zero where a genus has a significantly higher rate of taxonomic redescription than other genera.
(CSV)

S1 Code Raw data and analysis scripts for this project. This code is also available online at http://github.com/gaurav/aou_checklists and has been archived in Zenodo under DOI http://doi.org/10.5281/zenodo.1214826.
(ZIP)
