# Supplementary figures and images for: The tempo and mode of the taxonomic correction process: How taxonomists have corrected and recorrected North American bird species over the last 127 years
Source: PLoS One. 2018 Apr 19;13(4):e0195736. doi: 10.1371/journal.pone.0195736 (PMC5909608; doi:10.1371/journal.pone.0195736)

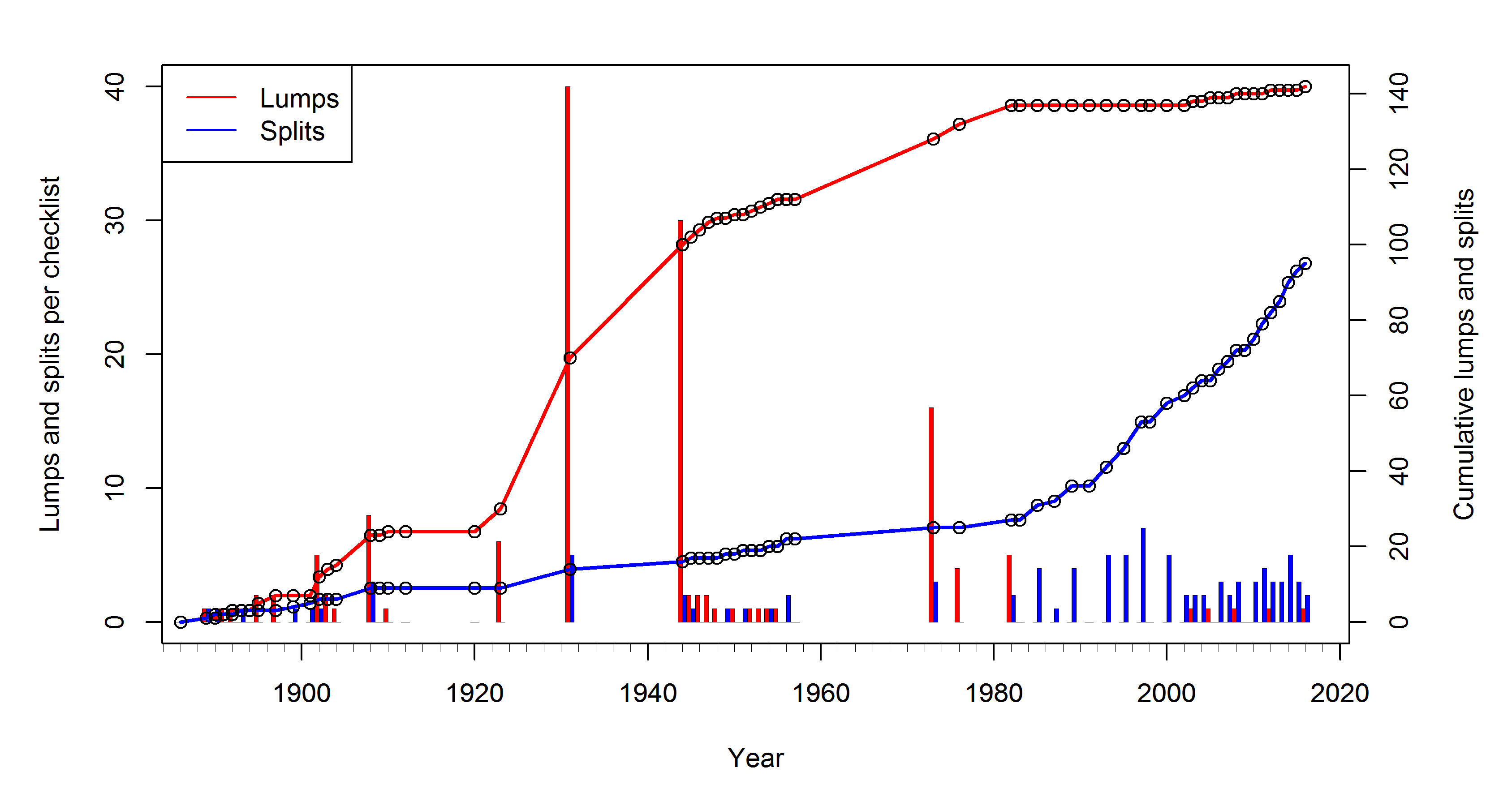

Supplement: S1 Code — This code is also available online at http://github.com/gaurav/aou_checklists and has been archived in Zenodo under DOI http://doi.org/10.5281/zenodo.1214826. (ZIP) [file pone.0195736.s007.zip › gaurav-aou_checklists-5955902/05 - analysis_by_year/graphs/pre1982_species/cumul_lumps_and_splits_bargraph.png]

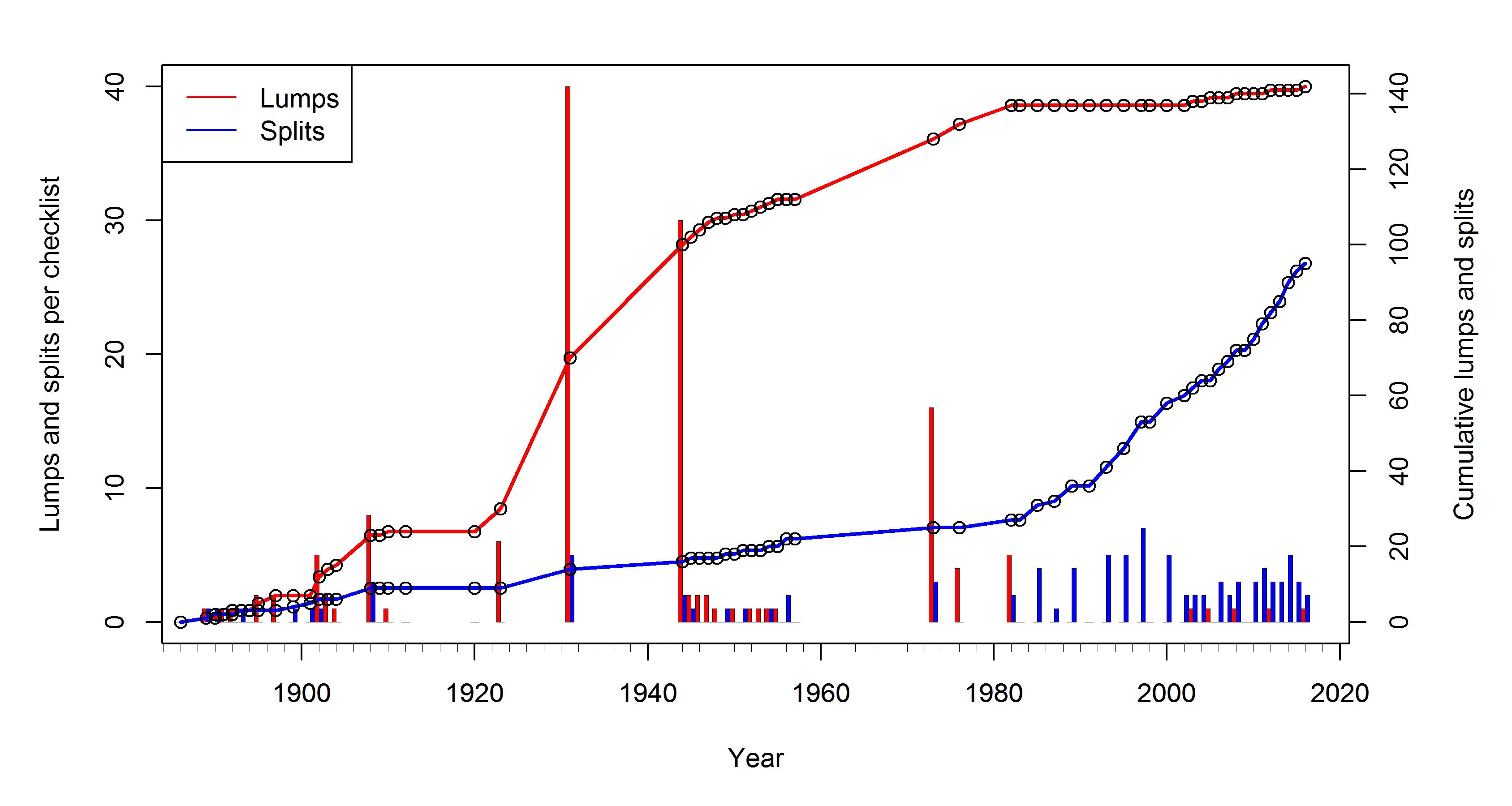

Supplement: S1 Code — This code is also available online at http://github.com/gaurav/aou_checklists and has been archived in Zenodo under DOI http://doi.org/10.5281/zenodo.1214826. (ZIP) [file pone.0195736.s007.zip › gaurav-aou_checklists-5955902/05 - analysis_by_year/graphs/pre1982_species/cumul_lumps_and_splits_bargraph.tif]

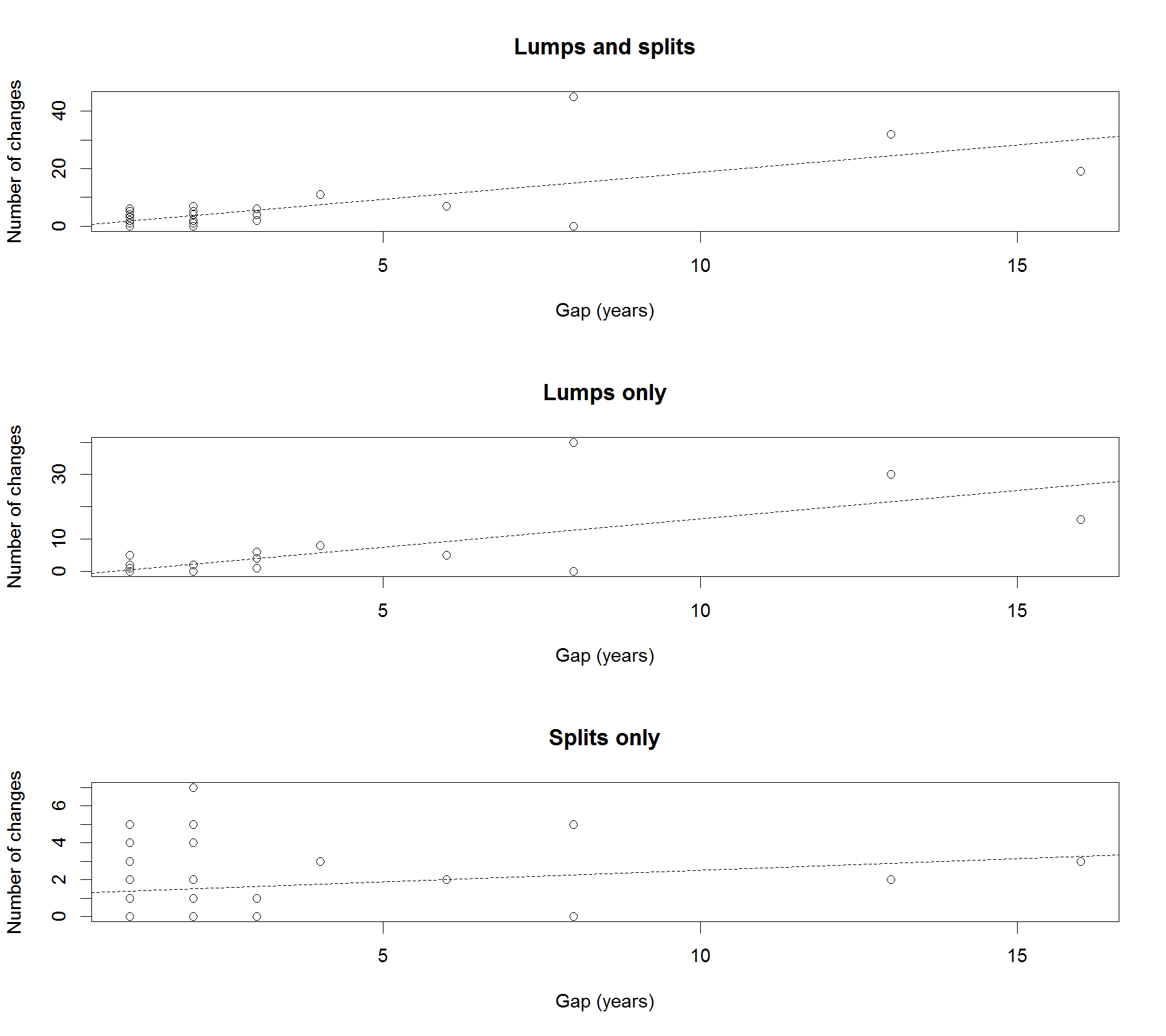

Supplement: S1 Code — This code is also available online at http://github.com/gaurav/aou_checklists and has been archived in Zenodo under DOI http://doi.org/10.5281/zenodo.1214826. (ZIP) [file pone.0195736.s007.zip › gaurav-aou_checklists-5955902/05 - analysis_by_year/graphs/pre1982_species/gaps.png]

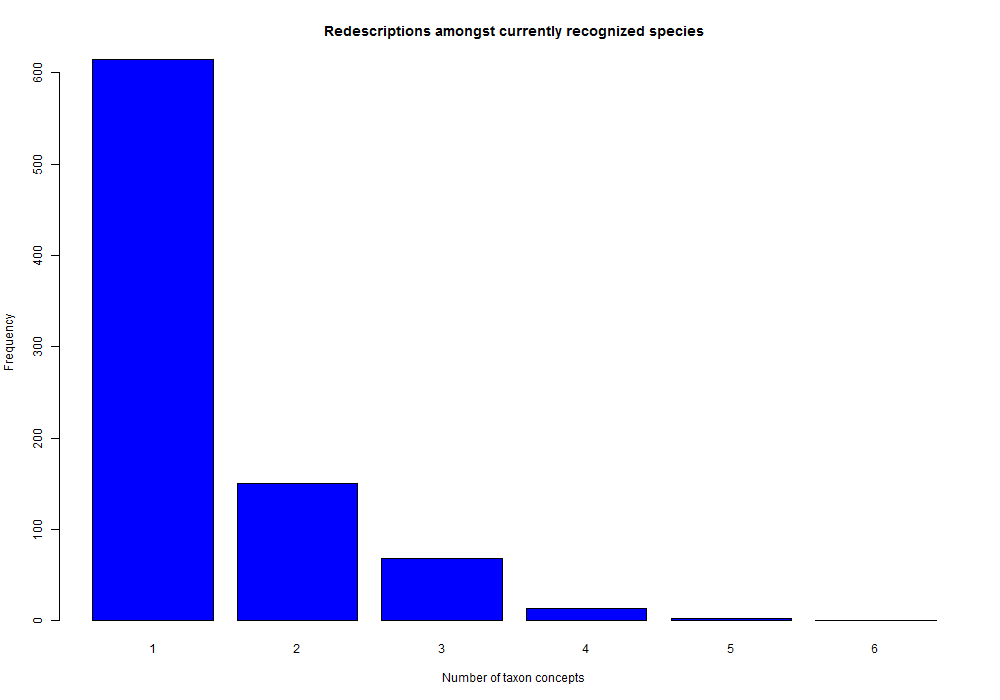

Supplement: S1 Code — This code is also available online at http://github.com/gaurav/aou_checklists and has been archived in Zenodo under DOI http://doi.org/10.5281/zenodo.1214826. (ZIP) [file pone.0195736.s007.zip › gaurav-aou_checklists-5955902/05 - analysis_by_year/graphs/pre1982_species/hist_definition_counts.png]

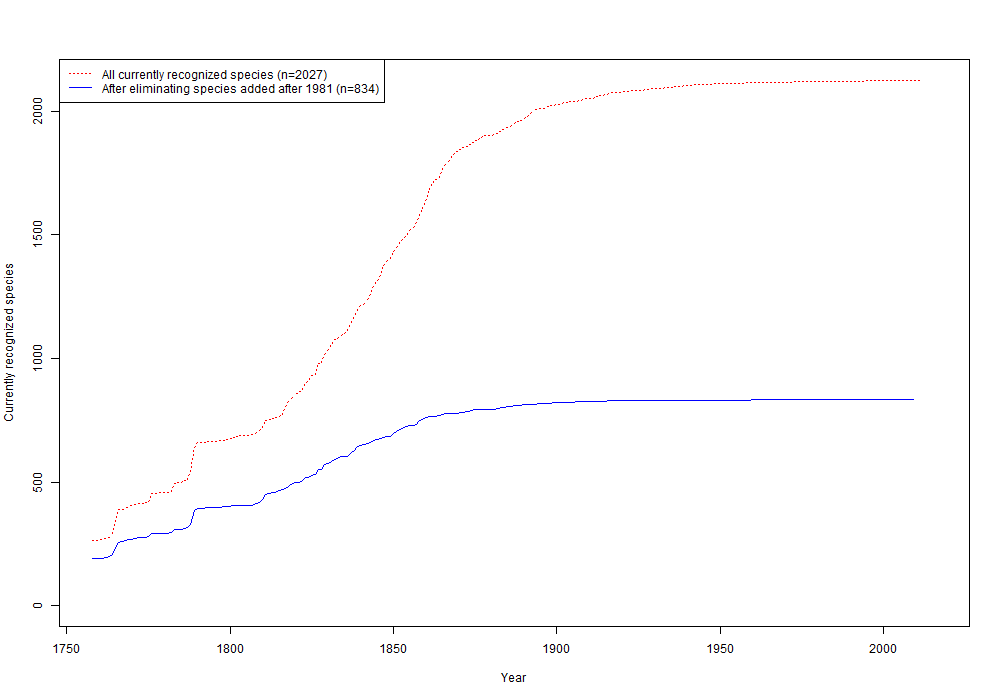

Supplement: S1 Code — This code is also available online at http://github.com/gaurav/aou_checklists and has been archived in Zenodo under DOI http://doi.org/10.5281/zenodo.1214826. (ZIP) [file pone.0195736.s007.zip › gaurav-aou_checklists-5955902/05 - analysis_by_year/graphs/pre1982_species/species_description_curves.png]

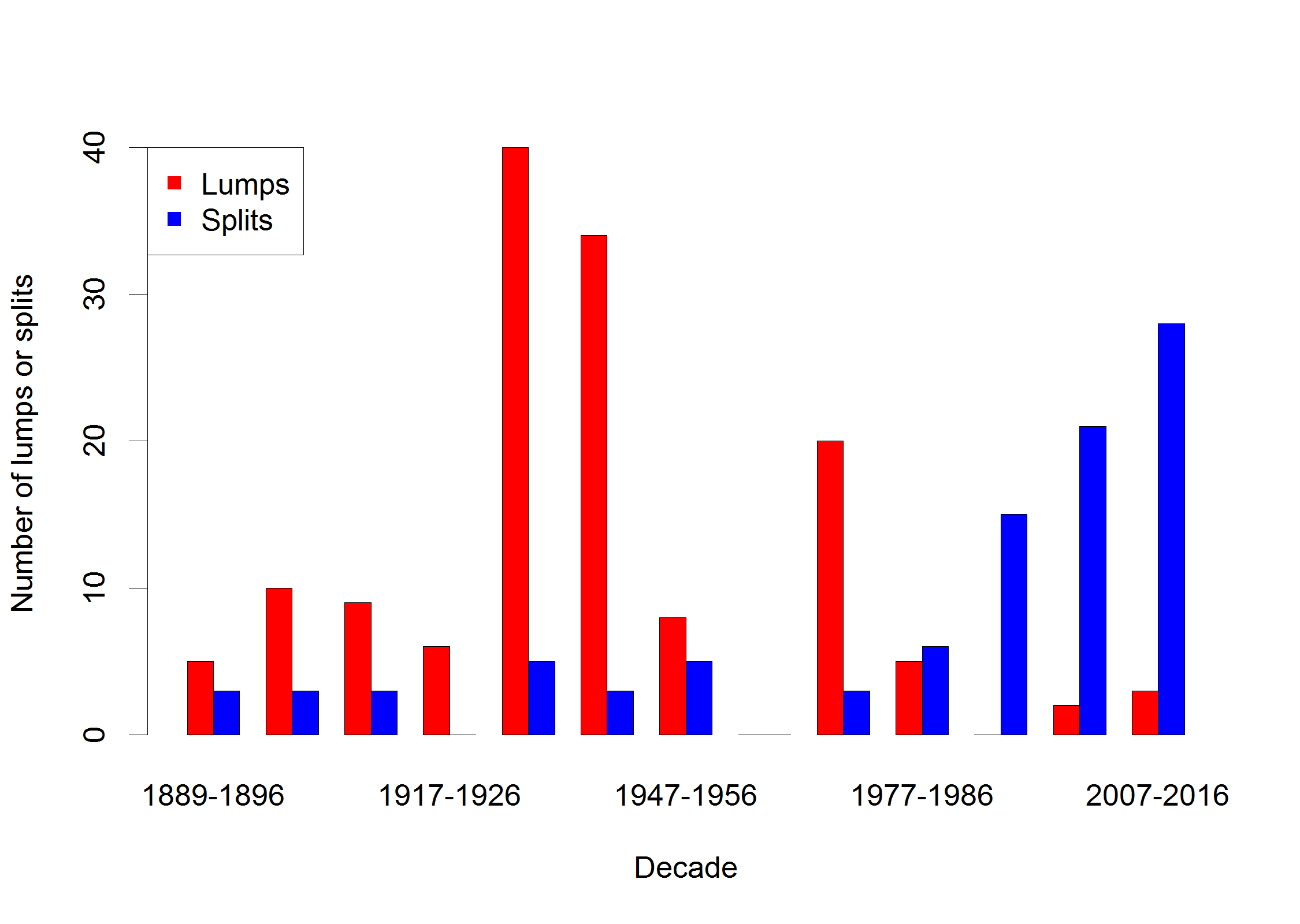

Supplement: S1 Code — This code is also available online at http://github.com/gaurav/aou_checklists and has been archived in Zenodo under DOI http://doi.org/10.5281/zenodo.1214826. (ZIP) [file pone.0195736.s007.zip › gaurav-aou_checklists-5955902/05 - analysis_by_year/graphs/pre1982_species/splumps_by_decade.png]

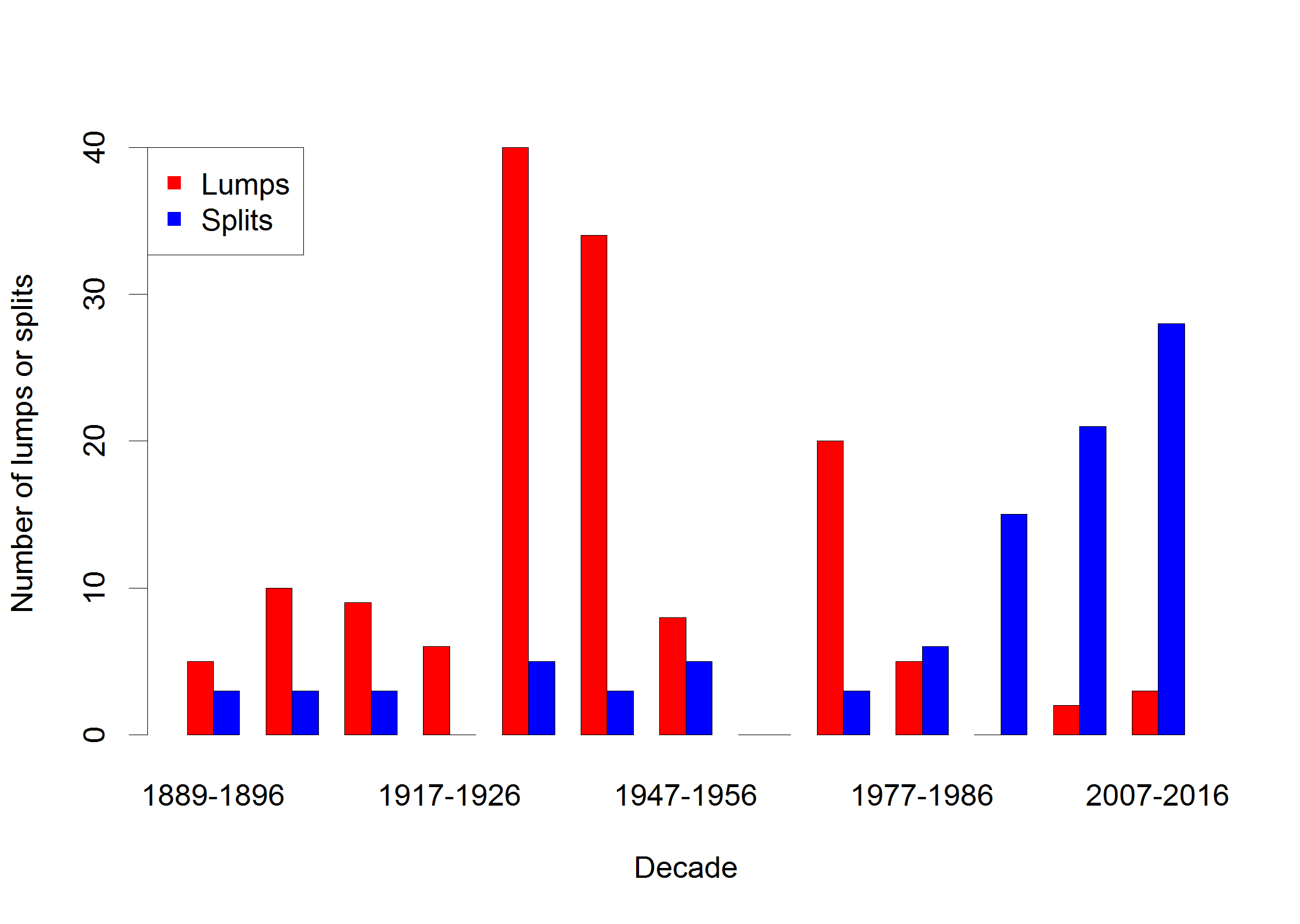

Supplement: S1 Code — This code is also available online at http://github.com/gaurav/aou_checklists and has been archived in Zenodo under DOI http://doi.org/10.5281/zenodo.1214826. (ZIP) [file pone.0195736.s007.zip › gaurav-aou_checklists-5955902/05 - analysis_by_year/graphs/pre1982_species/splumps_by_decade.tif]

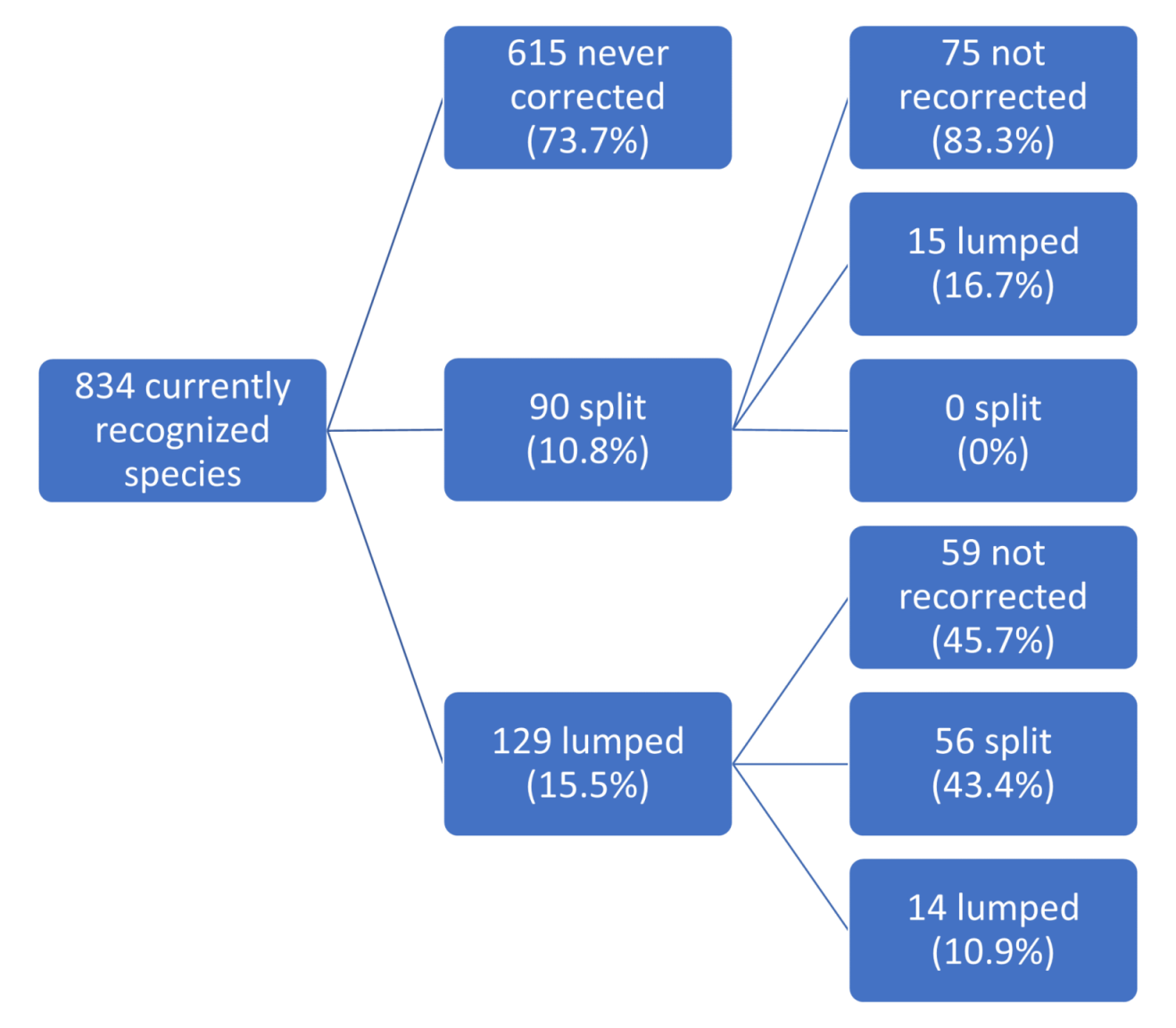

Supplement: S1 Code — This code is also available online at http://github.com/gaurav/aou_checklists and has been archived in Zenodo under DOI http://doi.org/10.5281/zenodo.1214826. (ZIP) [file pone.0195736.s007.zip › gaurav-aou_checklists-5955902/Diagram of recorrection patterns/Figure 3 - cropped.png]

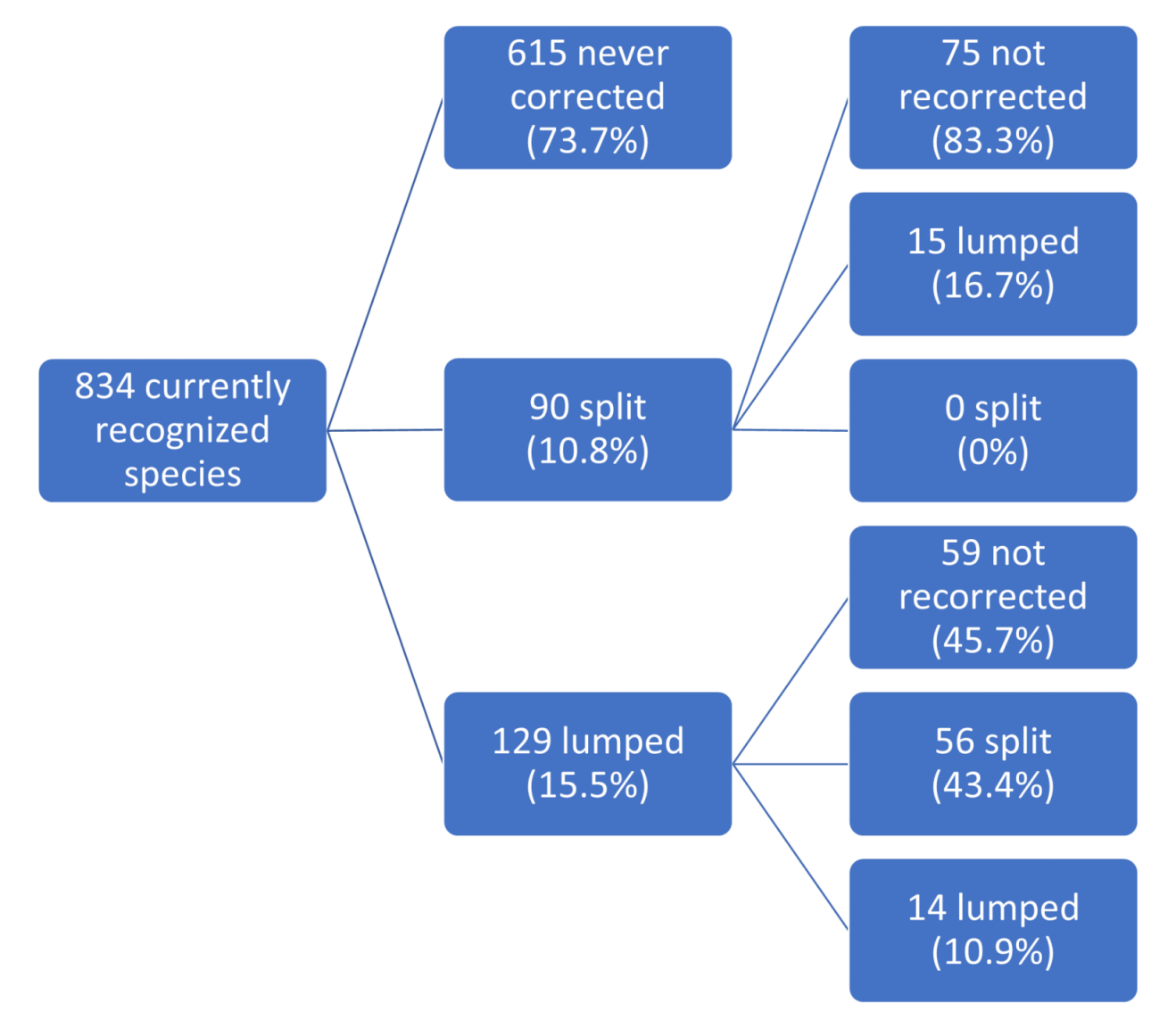

Supplement: S1 Code — This code is also available online at http://github.com/gaurav/aou_checklists and has been archived in Zenodo under DOI http://doi.org/10.5281/zenodo.1214826. (ZIP) [file pone.0195736.s007.zip › gaurav-aou_checklists-5955902/Diagram of recorrection patterns/Figure 3 - cropped.tif]

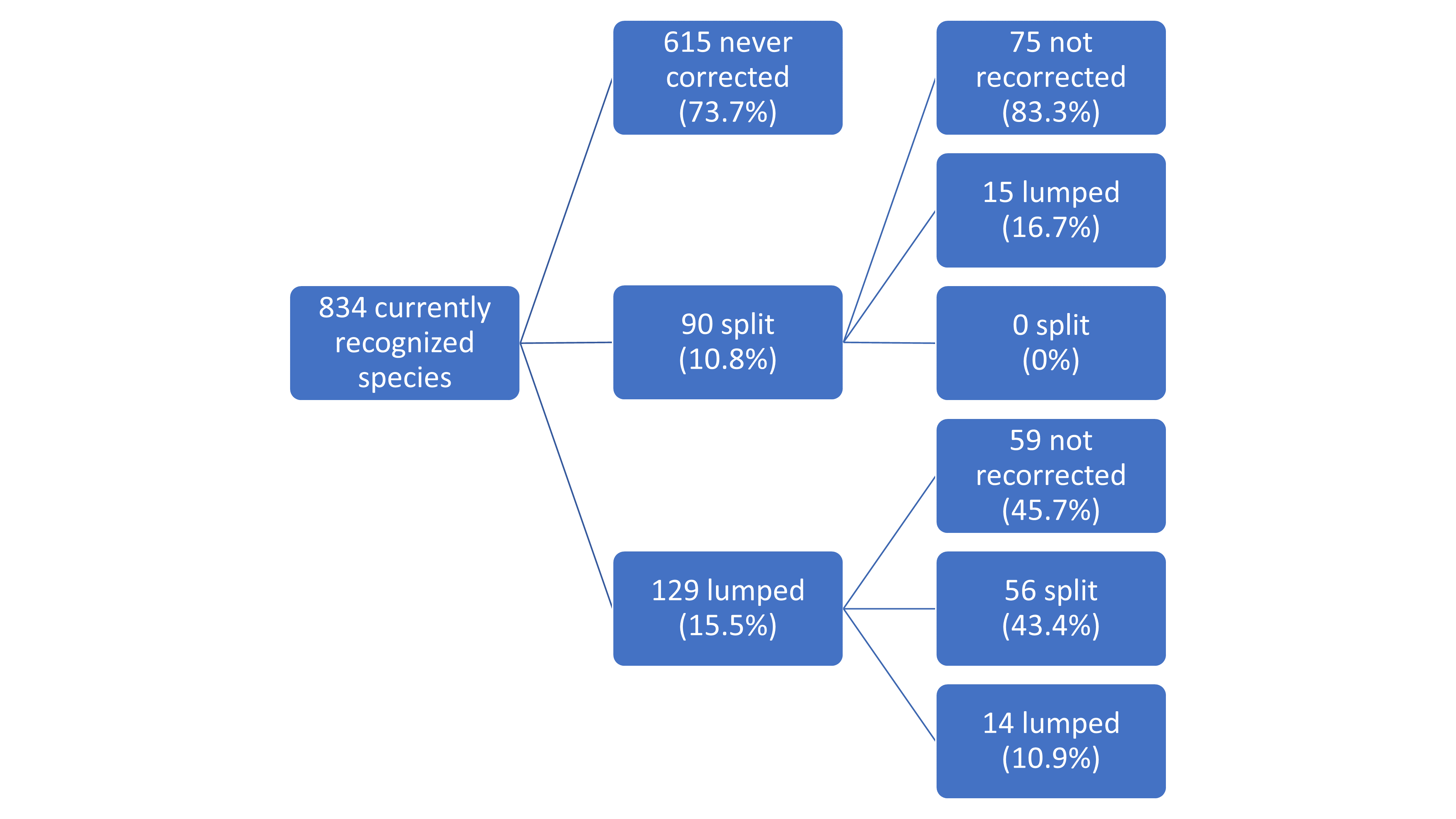

Supplement: S1 Code — This code is also available online at http://github.com/gaurav/aou_checklists and has been archived in Zenodo under DOI http://doi.org/10.5281/zenodo.1214826. (ZIP) [file pone.0195736.s007.zip › gaurav-aou_checklists-5955902/Diagram of recorrection patterns/Figure 3.png]

## Slide 1
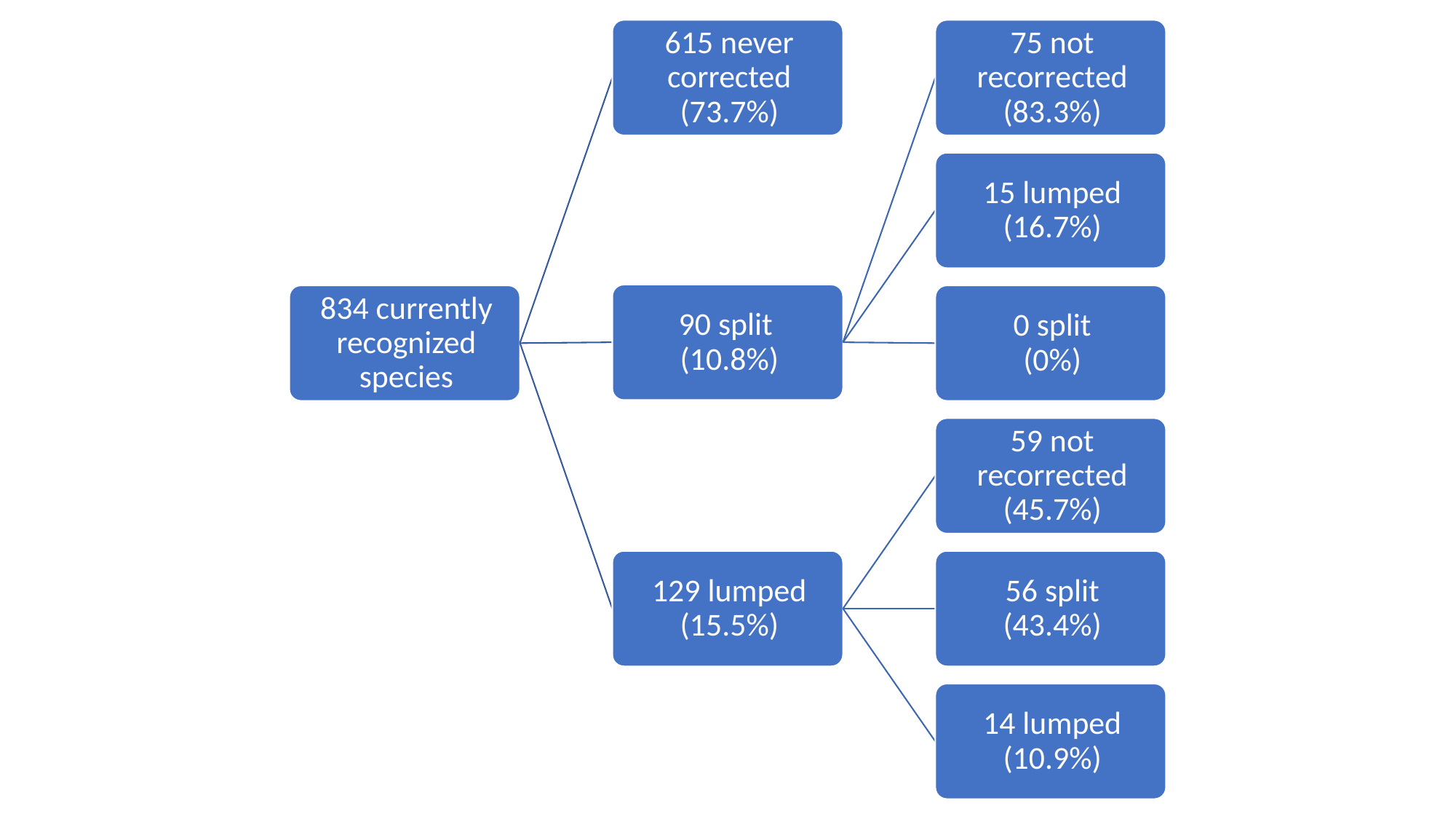

Supplement: S1 Code — This code is also available online at http://github.com/gaurav/aou_checklists and has been archived in Zenodo under DOI http://doi.org/10.5281/zenodo.1214826. (ZIP) [file pone.0195736.s007.zip › gaurav-aou_checklists-5955902/Diagram of recorrection patterns/Figure 3.pptx]

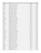

Supplement: S1 Code — This code is also available online at http://github.com/gaurav/aou_checklists and has been archived in Zenodo under DOI http://doi.org/10.5281/zenodo.1214826. (ZIP) [file pone.0195736.s007.zip › gaurav-aou_checklists-5955902/original_descriptions/previous_original_descriptions/descriptions as of September 2014/description_years (Numbers).numbers/preview-micro.jpg]

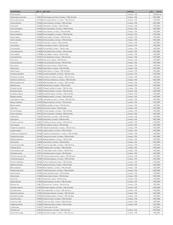

Supplement: S1 Code — This code is also available online at http://github.com/gaurav/aou_checklists and has been archived in Zenodo under DOI http://doi.org/10.5281/zenodo.1214826. (ZIP) [file pone.0195736.s007.zip › gaurav-aou_checklists-5955902/original_descriptions/previous_original_descriptions/descriptions as of September 2014/description_years (Numbers).numbers/preview-web.jpg]

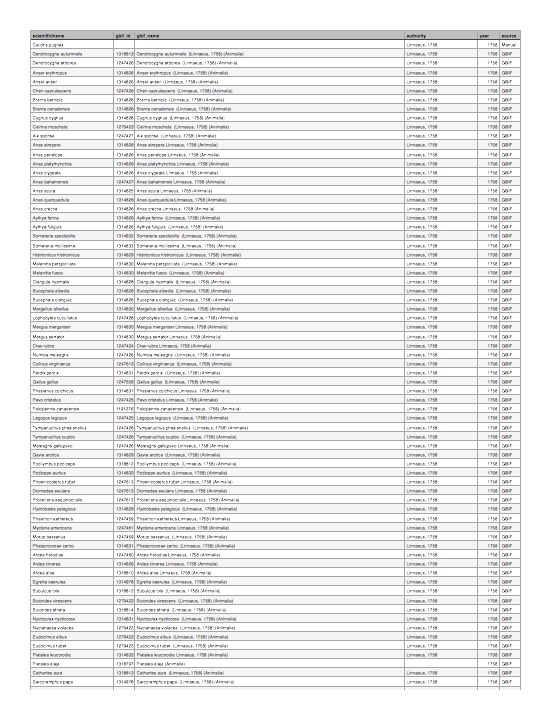

Supplement: S1 Code — This code is also available online at http://github.com/gaurav/aou_checklists and has been archived in Zenodo under DOI http://doi.org/10.5281/zenodo.1214826. (ZIP) [file pone.0195736.s007.zip › gaurav-aou_checklists-5955902/original_descriptions/previous_original_descriptions/descriptions as of September 2014/description_years (Numbers).numbers/preview.jpg]
